# PD103 Pediatric Exoskeleton For The Treatment Of Spinal Cord Atrophy And Other Neuromuscular Diseases

**DOI:** 10.1017/S0266462324003489

**Published:** 2025-01-07

**Authors:** Ana Alcalde Loeches, Blanca Novella Arribas, Gustavo Mora Navarro, Pilar Loeches Belinchon, Francisco Rodriguez Salvanés

## Abstract

**Introduction:**

Spinal cord atrophy (SMA) affects one in every 1,000 live births in Spain. Cerebral palsy (CP) is the most common cause of chronic motor disability in children, affecting two to three in every 1,000 live births. The Atlas 2030 exoskeleton is the first portable robotic pediatric exoskeleton aimed at facilitating walking in these children. It is used as a complement to other therapies.

**Methods:**

We aimed to compare the efficacy and safety of the ATLAS 2030 exoskeleton with conventional physiotherapy in children aged between four and 12 years with SMA or CP. We conducted a literature search to identify clinical trials and systematic reviews in the PubMed, Embase, Web of Science, and Cochrane Library databases, finding 201 original articles published between 2017 and 2022. No controlled studies were found on the ATLAS 2030 or any other portable device, even after expanding the searches to any robot-assisted gait devices.

**Results:**

Although systematic reviews evaluating the efficacy and safety of robotic orthoses in general were found, there were no specific controlled studies on the ATLAS 2030. The five studies found (two on CP and three on SMA) were of low quality and did not show conclusive results in terms of efficacy and safety. However, some of them suggested that this technology could provide significant improvements in acceptability, range of motion, and spasticity. With respect to safety there appeared to be no evidence of serious adverse effects from its use, reinforcing the developer’s initial hypothesis that the technology is safe.

**Conclusions:**

Although the available evidence is very limited, the ATLAS 2030 appears to show efficacy in some gait-related outcomes without serious adverse effects. To demonstrate safety and efficacy, randomized controlled trials are required that compare the ATLAS 2030 with conventional physiotherapy, with more participants and a longer duration, and are conducted by independent expert groups without conflicts of interest.

